# Rheumatoid factor, not antibodies against citrullinated proteins, is associated with baseline disease activity in rheumatoid arthritis clinical trials

**DOI:** 10.1186/s13075-015-0736-9

**Published:** 2015-08-26

**Authors:** Daniel Aletaha, Farideh Alasti, Josef S. Smolen

**Affiliations:** Division of Rheumatology, Department of Internal Medicine 3, Medical University Vienna, Waehringer Guertel 18-20, 1090 Vienna, Austria; 2nd Department of Medicine, Hietzing Hospital, Wolkersbergenstrasse 1, 1130 Vienna, Austria

## Abstract

**Introduction:**

Although the prognostic value of rheumatoid factor (RF) and autoantibodies against citrullinated proteins (ACPAs) in patients with rheumatoid arthritis (RA) is well established, their association with RA disease activity remains unclear. Here, we investigate this association in a large study using data from clinical trials.

**Methods:**

We used baseline data from four recent randomized controlled clinical trials of RA. We investigated individual and composite measures of disease activity. The relationship of RF and ACPAs with these measures was investigated by using stratified analysis (comparing four groups of patients according to the presence or absence of RF and ACPAs) and matched analysis (disease activity levels compared between patients negative and patients highly positive for one autoantibody who were matched for levels of the other autoantibody as well as for age, gender, and duration of RA).

**Results:**

A total of 2118 patients were analysed in the different cohorts. In the stratified analysis, RF^+^ patients, regardless of ACPA status, had the highest levels of disease activity, whereas ACPA^+^ patients had disease activity that was similar to or lower than that of ACPA^−^ patients, both in the presence and in the absence of RF. When matched for ACPA levels, patients with highly positive RF had significantly higher disease activity for all composite indices compared with patients who were RF^−^ (*P* = 0.0067), whereas ACPA-highly-positive and ACPA-negative patients matched for RF levels had similar disease activity, again even with the tendency toward lower disease activity for ACPA^+^ patients (*P* = 0.054).

**Conclusion:**

The data presented suggest that the presence of RF has a clear association with higher levels of disease activity but that the presence of ACPAs has not and even appears to be associated with lower disease activity.

**Electronic supplementary material:**

The online version of this article (doi:10.1186/s13075-015-0736-9) contains supplementary material, which is available to authorized users.

## Introduction

Rheumatoid arthritis (RA), a chronic and destructive inflammatory disease of the joints, is regarded as an autoimmune disease based on the presence of various autoimmune phenomena [1]. The most characteristic expression of the autoimmune response is the presence of autoantibodies, such as those directed to immunoglobulin G (rheumatoid factor, RF), citrullinated peptides (ACPA), or others [2, 3]. Depending on the stage of the disease, RF and ACPAs can be found in up to 75 % of patients with RA and occur concomitantly in about 80 % of the autoantibody-carrying ones [4]. The value of these antibodies is usually related to two important epidemiological aspects: diagnosis and prognosis. In this regard, they seem to have similar specificities [4–6], although several reports have suggested that ACPAs may be more specific and that the association of RF with progression of joint destruction may be driven mainly by the concomitantly present ACPAs [7, 8]; however, these observations have not found unequivocal agreement [4, 9, 10].

Prognosis usually relates to progression of disease, which is assessed by joint damage as a proxy. Progression in RA is mediated primarily by disease activity, as expressed by high swollen joint counts, elevated levels of acute-phase reactants, or composite measures of disease activity. RF has been shown to be linked primarily to joint damage via its association with disease activity, but a more direct and disease activity-independent effect on joint damage progression was also found, presumably because RF has direct effects on osteoclastogenesis and chondrocyte activation [11, 12].

Although the association of RF and ACPAs with disease progression is well investigated, their differential or independent association with disease activity is not clear. In the present study, we investigate whether the contribution of ACPAs and RF to disease activity is similar or related to one of the two. We use data from recent clinical trials in which both ACPAs and RF had been determined. Since the purpose is not to investigate therapeutic effects but rather the cross-sectional associations of RF and ACPAs with disease activity, we used baseline data of RA clinical trials, in which patients have active disease and their antibody status would not be influenced by any incoming therapeutic effect.

## Methods

### Patients and data

The trial sponsor kindly provided us with a 90 % random sample of patient level data from the multi-national, multi-centre IMAGE trial [13], in which the effects of a combination of methotrexate (MTX) plus rituximab at doses of 500 or 1000 mg, given as infusions 2 weeks apart, were compared with that of MTX monotherapy in MTX-naïve patients with RA. Since we used only baseline data of this trial, we combined the baseline data of all arms.

Another sponsor kindly provided us with a 90 % random sample of patient level data from several multi-national, multi-centre trials, in which the effects of two doses of golimumab (50 and 100 mg every 4 weeks applied subcutaneously), with or without MTX or other disease-modifying anti-rheumatic drugs, were compared with the effects of either newly applied methotrexate (in MTX-naïve patients) or placebo (in insufficient responders to prior MTX or other tumor necrosis factor (TNF) inhibitor therapies; Go-BEFORE, Go-FORWARD, and Go-AFTER trials) [14–16]. For the present evaluation, we mostly pooled the baseline data of all trial arms of all three studies; in one analysis, we separately assessed patients with early RA (Go-BEFORE) and established RA (Go-FORWARD and Go-AFTER).

The available data from all mentioned trials included demographic characteristics as well as clinical variables, such as swollen and tender joint counts (SJC and TJC), patient’s and physician’s global assessments, acute-phase response levels (C-reactive protein [CRP] and erythrocyte sedimentation rate [ESR]), the health assessment questionnaire disability index, and RF and ACPA levels. From the respective data, composite measures of disease activity—namely the Disease Activity Score using 28 joint counts (DAS28) using both ESR and CRP and the simplified and the clinical disease activity indices (SDAI and CDAI)—were calculated according to established formulae [17–19]. RF was measured centrally by using EL-RF/3 from TheraTest Laboratories (Lombard, IL, USA) and ACPAs by the Diastat enzyme-linked immunosorbent assay manufactured by Axis-Shield (Dundee, UK). Positivity, or not, of ACPAs and RF obviously will depend on the used test; false-negative classification of the serological status is therefore possible, particularly for ACPAs.

### Analyses

Our initial analysis was performed in the rituximab database, and additional validation studies were done in the golimumab dataset. We analysed the effects of RF and ACPAs on disease activity levels by using two different approaches: a stratified and a matched analysis.

#### Stratified analysis

We formed four subgroups according to the presence or absence of these autoantibody specificities (RF^−^/ACPA^−^, RF^+^/ACPA^−^, RF^−^/ACPA^+^, and RF^+^/ACPA^+^). After calculating the mean values of baseline demographic and disease activity variables, including composite indices, as well as baseline measures of physical function and joint damage, we assessed whether there were any differences across all groups by Kruskal-Wallis test. If significant differences were seen in this overall test, we performed pairwise comparisons between the groups by using Wilcoxon’s test. A *P* value of less than 0.05 was considered significant in all analyses.

#### Matched analysis

We compared baseline disease activity levels of RF-negative patients with those of RF-highly-positive ones while we matched for ACPA levels, age, and duration of RA. This procedure used propensity scores of the mentioned covariate variables to create 1:1 matches for patients tested negative versus high positive for RF (or ACPAs in the other analysis).

We used the Greedy matching algorithms and the Mahalanobis distance calculation [20]. This approach controlled for the effect of one serological marker (ACPAs), while the effect of the other (RF) could be investigated with a strong contrast (negative versus highly positive). Highly positive was defined as patients with RF levels greater than the median of all RF-positive patients. We repeated the analysis analogously by comparing ACPA-negative and ACPA-high-positive patients matched by RF levels, age, and duration of RA.

Given the high co-linearity of RF and ACPAs (approximately 80 % of seropositive patients are double-positive), we refrained from regression modelling in this study. Since patients at baseline differed regarding glucocorticoid use, we performed a subgroup analysis of users versus non-users regarding their SDAI levels in the rituximab database.

## Results

### Patient characteristics

Baseline demographic data and disease characteristics have been published previously for the individual arms and trials [13–16]. We have pooled arms of the rituximab IMAGE trial (n = 748) and the golimumab trials (n = 1370), and the baseline characteristics of these pooled populations are shown in Table 1.

**Table 1:** Patient characteristics

	Rituximab	Golimumab	All
Patients, number	748	1370	2118
Age, years	47.9 (13.1)	51.2 (12.2)	50.1 (12.6)
Disease duration, years	0.9 (1.2)	7.3 (8.1)	5.1 (7.2)
Female, %	81.2	81.8	81.5
Rheumatoid factor, % positive	87.4	75.4	79.7
ACPAs, % positive	86.0	73.6	78.0
Health assessment questionnaire	1.8 (0.7)	1.5 (0.)	1.6 (0.78)
DAS28-ESR	7.1 (1.0)	5.5 (1.0)	6.3 (1.3)
DAS28-CRP	5.7 (0.9)	5.1 (1.0)	5.3 (1.0)
SDAI	49.6 (14.8)	39.4 (14.6)	43.0 (15.5)
CDAI	46.4 (14.0)	37.2 (13.7)	40.5 (14.5)
Swollen joint count, 0–28	14.5 (6.4)	10.6 (6.0)	12.0 (6.4)
Tender joint count, 0–28	18.2 (7.1)	14.5 (7.41)	15.8 (7.5)
Pain, VAS in mm	63.5 (22.3)	63.0 (22.0)	63.2 (22.1)
Patient global assessment, VAS in mm	68.5 (21.5)	60.2 (23.4)	63.1 (23.1)
Evaluator global assessment, VAS in mm	68.2 (17.8)	60.7 (17.8)	63.3 (18.2)
ESR, mm	59.1 (29.5)	41.0 (26.3)	50.7 (29.5)
CRP, mg/dl	3.2 (2.9)	2.1 (2.9)	2.5 (2.9)
Radiographic score	7.5 (11.2)	25.8 (41.6)	17.8 (33.3)

*ACPA* antibody against citrullinated proteins, *DAS28-ESR* Disease Activity Score using 28 joint counts based on erythrocyte sedimentation rate, *DAS28-CRP* Disease Activity Score using 28 joint counts based on C-reactive protein, *SDAI* simplified disease activity index, *CDAI* clinical disease activity index, *VAS* visual analogue scale

As depicted in Tables 1, 2, and 3, there was a significant (about 75–85 %) overlap between the presence of RF and ACPAs. This broad overlap is well established, was already noted in early studies of ACPAs [3, 4], and is seen to a generally similar extent in our study. In the IMAGE trial, one of the entry criteria into the study was the presence of RF or, in its absence, the presence of erosions *and* elevated CRP levels [13]. In the golimumab trials, RF or ACPAs were required or, alternatively, damage and elevated CRP [14, 15].

**Table 2:** Patient demographics, disease activity, and functional and structural characteristics according to serological status of RF and ACPAs: rituximab database

	Patient subgroups according autoantibody status				*P* values of direct group comparisons	
	(1) RF^−^/ACPA^−^	(2) RF^−^/ACPA^+^	(3) RF^+^/ACPA^−^	(4) RF^+^/ACPA^+^	Overall	Pairwise comparisons
Patients, number	64	29	40	611	0.48	Not done
Age, years	52.0 (13.5)	49.2 (10.7)	46.1 (14.6)	47.5 (13.0)	0.05	Not done
Disease duration, years	1.0 (1.0)	0.7 (0.9)	0.8 (0.9)	1.0 (1.2)	0.58	Not done
Female, %	82.8	89.7	92.5	80.0	0.15	Not done
Rheumatoid factor, U/ml	<15	15.0 (0.0)	170 (199)	517 (854)	n.d.	Not done
ACPAs, U/ml	2.1 (0.3)	221 (270)	2.9 (1.1)	392 (660)	n.d.	Not done
Health assessment questionnaire	1.6 (0.6)	1.7 (0.7)	1.8 (0.6)	1.8 (0.7)	0.11	Not done
Disease activity score 28, ESR	6.9 (1.0)	6.6 (0.9)	7.1 (1.2)	7.1 (1.0)	**0.0025**	(1) vs. (4)*; (2) vs. (3)*; (2) vs. (4)**
Disease activity score 28, CRP	5.6 (0.8)	5.3 (0.8)	5.9 (1.0)	5.8 (0.9)	**0.01**	(2) vs. (3)**; (2) vs. (4)**
Simplified disease activity index	47.8 (15.0)	42.3 (12.7)	53.1 (16.5)	49.8 (14.7)	**0.0076**	(2) vs. (3)**; (2) vs. (4)**
Clinical disease activity index	45.0 (14.7)	39.7 (12.2)	49.9 (15.5)	46.6 (13.8)	**0.0097**	(2) vs. (3)**; (2) vs. (4)**
Swollen joint count	14.2 (6.6)	11.7 (5.9)	16.7 (6.5)	14.5 (6.3)	**0.0094**	(2) vs. (3)**; (3) vs. (4)**; (2) vs. (4)**
Tender joint count	18.0 (7.0)	14.8 (5.6)	19.4 (8.1)	18.4 (7.1)	**0.02**	(2) vs. (3)*; (2) vs. (4)**
Pain, VAS	58.9 (21.2)	60.9 (21.5)	61.8 (22.1)	64.2 (22.5)	0.15	Not done
Patient global assessment, VAS	64.3 (19.3)	65.0 (21.6)	68.4 (21.6)	69.0 (21.7)	0.12	Not done
Evaluator global assessment, VAS	64.1 (20.0)	67.2 (12.3)	69.6 (16.3)	68.6 (17.9)	0.35	Not done
ESR	47.5 (25.4)	51.4 (29.0)	54.2 (29.1)	61.0 (29.5)	**0.0009**	(1) vs. (4)***
CRP	2.7 (2.6)	2.6 (2.0)	3.2 (3.5)	3.3 (2.9)	0.23	Not done
Radiographic score	5.2 (7.9)	7.1 (8.3)	5.77 (11.7)	7.92 (11.6)	**0.0066**	(1) vs. (4)*; (3) vs. (4)**

*n.d.* not determined, *ACPA* antibody against citrullinated proteins, *ESR* erythrocyte sedimentation rate, *CRP* C-reactive protein, *VAS* visual analogue scale

*P < 0.05; **P < 0.01; ***P < 0.001; bold P-values indicate significance at the <0.05 level. For these variables pairwise comparisons ensued

**Table 3:** Patient demographics, disease activity, and functional and structural characteristics according to serological status of RF and ACPA: golimumab database

	Patient subgroups according to autoantibody status				*P* values of direct group comparisons	
	(1) RF^−^/ACPA^−^	(2) RF^−^/ACPA^+^	(3) RF^+^/ACPA^−^	(4) RF^+^/ACPA^+^	Overall	Pairwise comparisons
Patients, number	268	68	93	937	0.48	Not done
Age, years	51.7 (12.8)	48.1 (14.4)	52.2 (12.0)	51.2 (11.9)	0.15	Not done
Disease duration, years	6.8 (8.5)	4.9 (5.7)	6.4 (6.9)	7.8 (8.2)	**<0.001**	(1) vs. (4)**; (2) vs. (4)***
Female, %	82.1	80.9	86.0	81.4	0.74	Not done
Rheumatoid factor	9.3 (2.8)	10.8 (2.6)	96 (162)	190 (275)	n.d.	Not done
ACPA	2.1 (0.4)	112 (167)	2.5 (0.9)	225 (409)	n.d.	Not done
Health assessment questionnaire	1.5 (0.6)	1.3 (0.7)	1.4 (0.7)	1.5 (0.7)	**0.02**	(1) vs. (2)*; (2) vs. (4)*
Disease activity score 28 (ESR)	5.6 (1.0)	5.1 (1.1)	5.6 (0.8)	5.5 (1)	0.06	Not done
Disease activity score 28 (CRP)	5.1 (1.0)	4.7 (1.0)	5.1 (1.0)	5.1 (1)	**0.007**	(1) vs. (2)***; (2) vs. (3)*; (2) vs. (4)**
Simplified disease activity index	40.7 (14.8)	33.4 (12.4)	40.0 (16.3)	39.3 (14.3)	**0.002**	(1) vs. (2)***; (2) vs. (3)*; (2) vs. (4)***
Clinical disease activity index	38.7 (14.1)	31.7 (12.2)	37.6 (14.9)	37.1 (13.4)	**0.002**	(1) vs. (2)***; (2) vs. (3)*; (2) vs. (4)***
Swollen joint count	10.7 (6.4)	8.7 (4.7)	10.9 (6.4)	10.7 (5.9)	0.06	Not done
Tender joint count	15.7 (7.9)	11.9 (6.9)	15.1 (8.0)	14.3 (7.2)	**0.001**	(1) vs. (2)***; (1) vs. (4)**; (2) vs. (3)*; (2) vs. (4)**
Pain (VAS)	65.2 (19.5)	61.1 (25.0)	60.4 (23.6)	62.7 (22.3)	0.41	Not done
Patient global assessment (VAS)	62.2 (22.1)	57.0 (25.0)	59.3 (24.7)	59.9 (23.5)	0.43	Not done
Evaluator global assessment (VAS)	60.7 (17.4)	53.6 (19.1)	57 (18.1)	61.5 (17.6)	**0.003**	(1) vs. (2)**; (3) vs. (4)*; (2) vs. (4)**
Erythrocyte sedimentation rate	37.1 (25.7)	38.0 (22.7)	40.1 (26.4)	42.8 (26.8)	0.08	Not done
C-reactive protein	1.9 (2.8)	1.8 (2.5)	2.3 (4.4)	2.2 (2.7)	**<0.001**	(1) vs. (4)***; (3) vs. (4)*; (2) vs. (4)*
Radiographic score	15 (26)	24.3 (47.2)	18.3 (31.7)	29.6 (44.9)	**<0.001**	(1) vs. (4)***; (3) vs. (4)*

*n.d.* not determined, *ACPA* antibody against citrullinated proteins, *ESR* erythrocyte sedimentation rate, *CRP* C-reactive protein, *VAS* visual analogue scale

*P < 0.05; **P < 0.01; ***P < 0.001; bold P-values indicate significance at the <0.05 level. For these variables pairwise comparisons ensued

### Stratified analysis

**Fig. 1:**
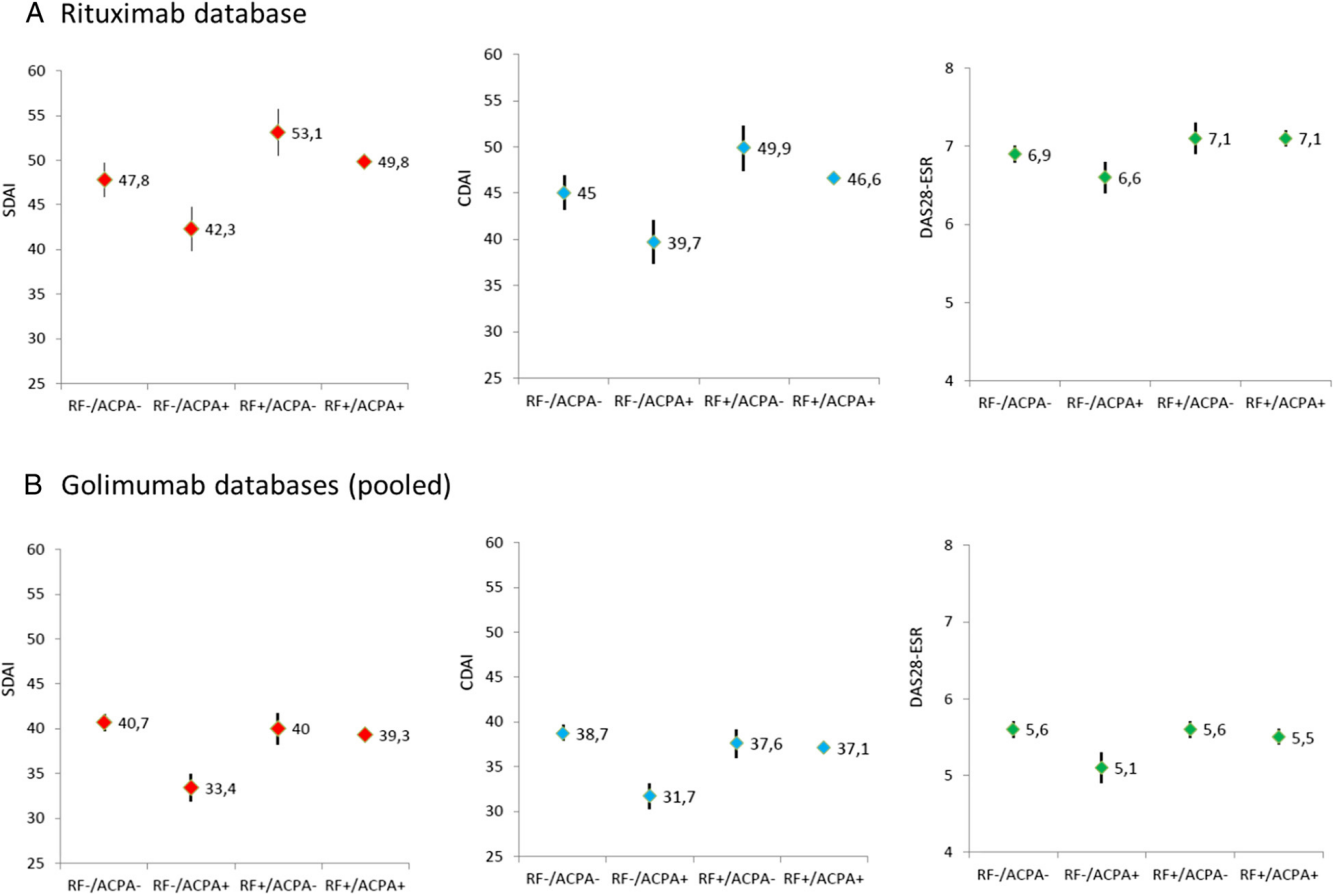
Disease activity at baseline of clinical trials stratified by the presence or absence of rheumatoid factor (RF) or anti-citrullinated peptide antibodies (ACPAs). **a** Rituximab database (IMAGE trial of early RA). **b** Golimumab database (several trials on RA patients with various disease durations). Please see Table 2 for details on numbers and statistics. *CDAI* clinical disease activity index, *DAS28-ESR* Disease Activity Score using 28 joint counts-erythrocyte sedimentation rate, *RA* rheumatoid arthritis, *SDAI* simplified disease activity index

In the initial analysis, we evaluated the IMAGE trial data by using baseline demographic, disease activity, functional, and radiographic values, stratified according to the presence or absence of both RF and ACPA. There were significant differences among the groups regarding composite measures of disease activity (Fig. 1a) as well as many disease activity-related variables. The overall *P* values are displayed in the column “overall” in Tables 2 and 3. Only if these overall tests were significant, we moved on to assess the group-wise comparisons. Interestingly, the lowest disease activity levels among all groups (Table 2, highlighted in bold) were seen for patients who had ACPAs as their only autoantibody. In contrast, the highest scores were generally found in double-positive patients or patients positive for only RF but not ACPAs, indicating that the presence of RF may be importantly related to higher disease activity but that ACPAs may not.

Indeed, RF^+^/ACPA^−^ (and also RF^+^/ACPA^+^) compared with RF^−^/ACPA^+^ patients had significantly higher levels of DAS28, SDAI, CDAI (Fig. 1), and also swollen and tender joint counts (Table 2). Moreover, in double-positive patients, the autoantibody levels were much higher than the level of RF or ACPAs in single-positive patients (Tables 2 and 3).

When we repeated these analyses in the golimumab database, in which patients with various disease durations and a broad range of medication histories were comprised (including failure of TNF inhibitors), comparable results were obtained: indeed, as shown in Fig. 1b and Table 3, the lowest disease activity was again observed in the RF^−^/ACPA^+^ group of patients, and significantly higher disease activity was found in RF^+^/ACPA^−^ (and even double-negative) patients for composite measures and joint counts (Table 3). Similar to the rituximab database, high levels of disease activity were seen in double-positive patients, which, as before, appeared driven primarily by RF, given the higher disease activity in RF^+^/ACPA^−^ patients compared with RF^−^/ACPA^+^ ones. Moreover, the differences in acute-phase reactant levels became even more apparent in the golimumab database, and likewise differences in physical function. Like in the rituximab database, autoantibody levels in double-positive patients were clearly higher compared with single-positive ones.

### Matched analysis

**Fig. 2:**
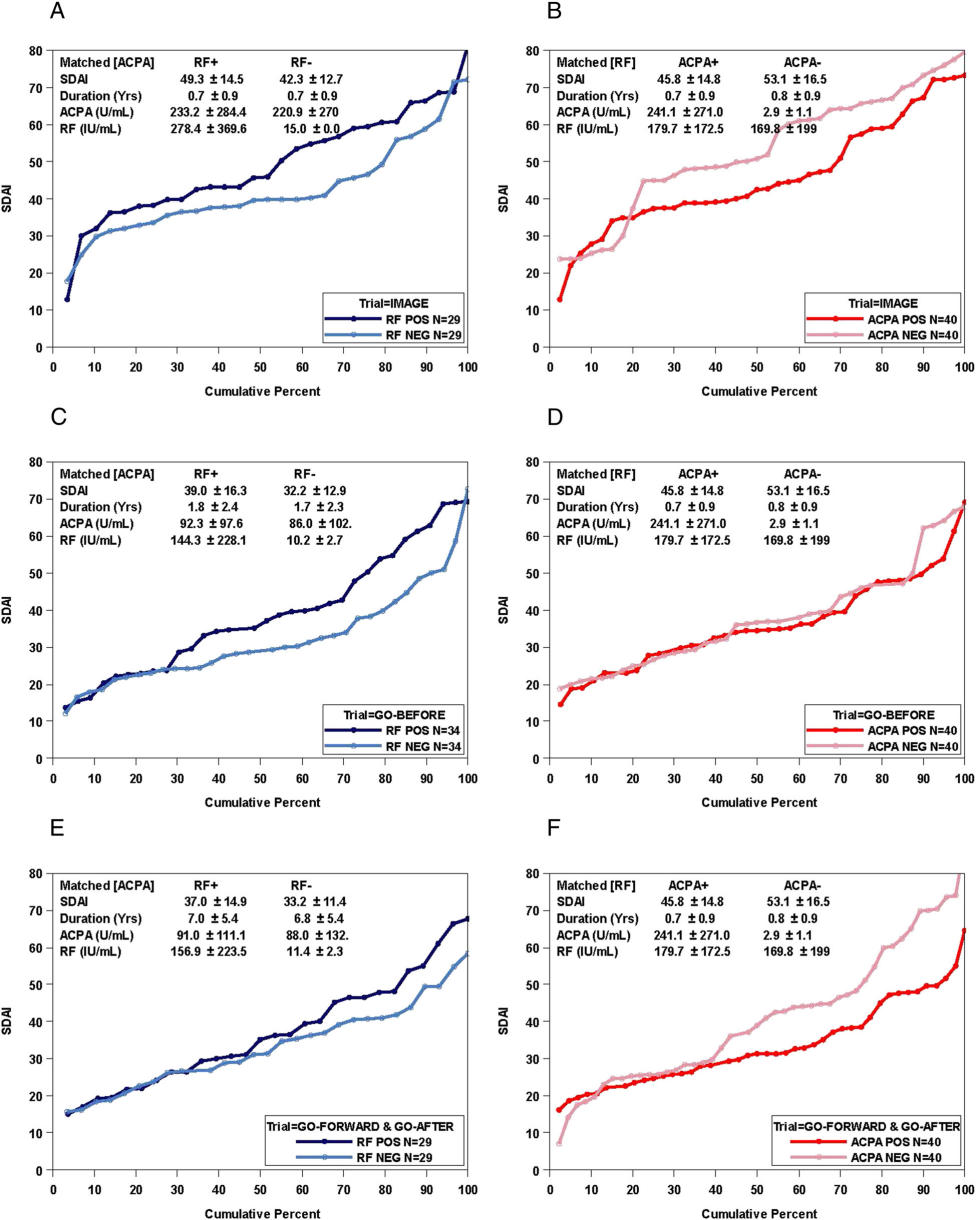
Disease activity distributions for the simplified disease activity index (SDAI) at baseline according to rheumatoid factor (RF) or anti-citrullinated peptide antibodies (ACPAs) or both. Probability plots of levels of disease activity according to the SDAI. **a**, **c**, **e** Distributions of baseline SDAI values by RF status (negative versus high positive) in patients matched for ACPA, age, and duration (higher disease activity in RF-positive patients; overall *P* = 0.0067). **b**, **d**, **f** Distributions of baseline SDAI values by ACPA status (negative versus highly positive) in patients matched for RF levels, age, and duration of RA (higher disease activity in ACPA-negative patients; overall *P* = 0.054). Data are separated by trials for IMAGE trial (a, b), GO-BEFORE (c, d), and combined GO-FORWARD+GO-AFTER (e, f)

Because of the large overlap of seropositivity for these two antibodies, and in order to control for the potential effect of autoantibody levels (as indicated above), we matched RF^−^ and RF-high^+^ patients for ACPA levels and found that the RF^+^ group had significantly higher disease activity than the RF^−^ group (Fig. 2, left panel inserts). In contrast, when we matched ACPA^−^ and ACPA-high^+^ patients for RF levels, we found higher disease activity in the ACPA^−^ compared with the ACPA^+^ patients (Fig. 2, right panel inserts). These data further supported the notion that higher disease activity was associated with the presence of RF and that ACPAs, if present, are associated with lower RA disease activity (Fig. 2b, d, f).

### Stratification by glucocorticoid use at baseline

When we compared glucocorticoid users versus non-users in the rituximab database regarding their SDAI values, there was no difference between the effects of RF or ACPA status on the ranking of average disease activity levels (Additional file 1: Table S1; SDAI results also presented in Additional file 1: Figure S1).

## Discussion

Disease activity is the major determinant of impairment of physical function and progression of joint damage [21, 22]. Recognizing elements that influence disease activity, therefore, is of particular importance. Autoantibodies have been suggested to increase disease activity by virtue of immune complex formation with or without complement activation through binding to Fc or complement receptors (or both) and subsequent increases in production of pro-inflammatory cytokines [12, 23]. In the present study, we have shown that baseline disease activity of patients with RA in clinical trials is positively associated with the presence of RF but not ACPA. This conclusion is derived from cross-sectional analyses of clinical trial patients; all of these patients had active disease and came from many centres in many countries and regions. They therefore constitute one of the broadest representations of RA patients in which such associations have been studied.

Interestingly, aside from the lower levels of composite measures of RA disease activity, RF^−^/ACPA^+^ patients had significantly lower levels across most of the individual clinical and laboratory disease activity variables and physical function compared not only with double-positive patients but also with those who were RF^+^/ACPA^−^. In some instances, ACPA^+^/RF^−^ patients even had lower scores than double-negative patients.

On the other hand, similar levels of disease activity were observed in RF^+^/ACPA^−^ compared with double-positive patients, despite the mentioned higher level of RF in the double-positive group. This may indicate either that the presence of RF, rather than its level, is the driving force for disease activity or alternatively that there is an effect of RF levels plus some “protective” effect of ACPA reducing higher disease activity due to higher RF levels in double-positives, therefore causing an apparent similarity of disease activity in these two groups. The latter hypothesis is clearly supported by two facts, the first of which is the comparison of ACPA^+^ and ACPA^−^ patients who are seronegative for RF. Here, in the absence of RF, the presence of ACPAs is also associated with lower levels of disease activity; the second fact is the matched analyses, which controlled for the level of the respective other autoantibody.

The important role of RF, and not of ACPA, in relation with higher levels of disease activity, was also implied in another recent study, in which the authors have revealed that RF^+^ patients regardless of ACPA status compared with RF^−^/ACPA^+^ patients exhibited not only higher disease activity but also higher levels of pro-inflammatory cytokines [24]. However, in that study, it was also hypothesized that ACPAs have a synergistic effect in the presence of RF regarding disease activity, a finding which can be neither supported nor ruled out by the present study.

Based on the epidemiological findings of our study, some translational explanations can be used to understand the clear relationship of RF with disease activity as well as its absence for ACPAs. Firstly, as assessed in routine practice and clinical trials, RF is of IgM isotype whereas ACPAs are mostly IgG antibodies; IgM isotype activates complement to a much larger extent than IgG [25], thus potentially leading to a stronger secondary inflammatory response via inflammatory complement breakdown products or complement-receptor-mediated macrophage activation or both [26–28]. In this setting, ACPAs might preferentially activate inhibitory Fcγ-receptors and thus mitigate the inflammatory response [29]. At the same time, however, it is conceivable that IgM ACPAs also exist but that current tests are able to detect IgG only.

Secondly, ACPAs likely represent products of long-lived memory plasma cells [30], whereas RF may be at least partly produced by a particular subset of B lymphocytes, B-1 cells; the former may have less plasticity and responsiveness to variations of the disease process or might be less involved in cell-cell interactions in RA than the latter [31, 32]. However, it should be noted that the existence of B-1 cells in humans has been a matter of some debate, although evidence of their existence is increasing [33–35] and they have been related especially to RF production in humans long ago [36]. However, here again epidemiological findings support their existence, as RF levels may change substantially in patients responding to therapies, but ACPA levels do not [37, 38]. This notion is reinforced by the high correlation of the presence of RF with comorbidity and cardiovascular death [39, 40]. Also, RF has been shown to induce pro-inflammatory cytokines like TNF [41]. All of these findings together support concepts that RF, and not ACPAs, is the main serological inflammatory player but that ACPAs on their own not only may not fuel RA disease activity but even may mitigate it. This finding should be clearly distinguished from the well-established association of ACPAs with radiographic damage [4]. Moreover, ACPAs have been suggested to have a direct effect on osteoclastogenesis, and therefore their association with joint damage may be independent of disease activity [42].

The broad overlap of RF and ACPAs has been a practical limitation for any study investigating differential effects of each of these antibodies and is also a limitation of our study. Indeed, the small sample size of single seropositive patients in the stratified comparisons may put the analysis at risk for incorrectly failing to reject the null hypothesis of no association of antibodies with disease activity. Since our results were statistically significant, a potentially limited sample size is obviously not relevant. In addition, we took an unprecedented approach to account for this co-linearity of RF and ACPAs by the above-matched analysis. Finally, the initial results obtained in one trial dataset were confirmed in a totally unrelated second large clinical trial database.

Since the data are based on clinical trials, another potential limitation may be selection bias related to the typical inclusion and exclusion criteria. This selection bias might affect the generalisability of the results. Particularly, results cannot be generalised to patients who have very little or no disease activity but in whom these associative analyses with disease activity would not be possible for lack of active disease. In all trials, some serological criteria *and* some minimal disease activity requirements applied. However, these two were always independent and never conditional. For the IMAGE trial, RF seronegativity at baseline was allowed only if there was evidence of erosive disease. Since ACPAs were not among the inclusion criteria in IMAGE, the prevalence of the autoantibodies (sizes of the four groups) is not necessarily reflective of a general RA population. We can nevertheless make the points for the validity of the results in the IMAGE trial by using the following arguments: firstly, in golimumab trials, ACPAs or RF (or both) were required, thus confirming the data of the IMAGE trial in another trial with different serological inclusion requirements; secondly, patients negative for RF were required to have elevated CRP and joint damage, thus biasing ACPA^+^/RF^−^ and ACPA^−^/RF^−^ patients toward higher rather than lower disease activity compared with the respective RF^+^ groups. The opposite was observed. That this was the reverse with the ACPA^+^/RF^−^ population further supports our conclusion, while the comparably high activity seen in double-negative patients may indeed be due to the—independent—prerequisite to have higher levels of disease activity. Notably, compared with double-positive patients, single-positive patients had lower levels of that respective autoantibody (about 50 % less), suggesting that the presence of both autoantibodies may be related to a higher autoimmune thrust than the presence of just one. Also, disease activity of RA is inherently fluctuant and, therefore, assessing it at a single time point, as analysed here, may not provide the full picture; however, the large group of patients studied here inherently has an averaging effect and, in addition, patients in clinical trials have to present with active disease at a pre-screening visit as well as the baseline visit, ensuring some degree of persistence in disease activity. Furthermore, patients in clinical trials come from many centres throughout many regions, thus reducing the potential selection bias that may be inherent to studies of patients coming from a single or just few centres. Finally, many of the potential differences were accounted for by the additional matched analysis.

The cross-sectional character of our study may be regarded as a limitation because antibody levels may change over time, particularly under the influence of effective therapy, although ACPA levels fluctuate less than RF levels [38]. However, the purpose here was to look at associations with autoantibody status and not with changes during therapy. We intentionally focused only on baseline data in order to prevent confounding by treatment. Also, in a separate analysis, glucocorticoid use at baseline did not have an influence on the observed results. In addition, the similarity of the results in the different trial populations, capturing MTX-naïve patients with early RA on the one hand (rituximab database) and patients across all lengths of disease duration and prior drug experience (golimumab database) on the other, was highly confirmatory and clearly adds to the generalisability of our findings. It is nevertheless important to bear in mind that, although the present data came from two large independent databases, analyses from additional databases in independent studies will be needed to fully confirm our conclusions.

## Conclusions

The presence of RF appears to have a clear association with higher levels of disease activity, whereas the presence of ACPAs is associated with lower disease activity, although this was observed only as a trend. The data further imply that therapeutic strategies in RA should focus not only on classic variables of disease activity but also on the presence of RF. As seroconversion into an autoantibody-negative state is attainable for RF, this should be a therapeutic goal, and lack of seroconversion a potentially decision-driving situation. In contrast, seroconversion has not yet been frequently seen for ACPAs [38, 43]. ACPAs, on the other hand, should be considered regarding structural outcomes, on which they may have an effect independent of disease activity.

We consider several relevant points of our findings for the practicing rheumatologist. Firstly, the data reveal that RF determination is important, and the presence of RF is related to higher levels of disease activity. Secondly, the data reveal that aside from their diagnostic value as detailed in the new American College of Rheumatology-European League Against Rheumatism (ACR-EULAR) classification criteria as well as their prognostic value regarding structural progression, ACPAs are not of major relevance regarding the risk of a highly active disease process (which is known to drive progression). Thirdly, the large overlap with RF positivity questions the value of determining ACPAs in the course of the disease, making it potentially relevant only for RF-negative patients or low-titre RF-positive patients, as previously suggested [4, 44]. Given the data at hand, only determining ACPAs without assessing RF, as has also been proposed by some [45], may be an unjustified approach.

## Additional file

Additional file 1: Figure S1. Disease activity at baseline in the rituximab database (IMAGE trial of early rheumatoid arthritis). Stratified analysis by the presence or absence of rheumatoid factor (RF) and of anti-citrullinated peptide antibodies (ACPA) (x-axis categories) as well as by the use, or not, of glucocorticoids. *SDAI* simplified disease activity index. **Table S1**. Disease activity indices at baseline in subgroups by RF and ACPA positivity, as well as by glucocorticoid use, or not. *ACPA* antibody against citrullinated peptides, *CDAI* clinical disease activity index, *DAS* Disease Activity Score, *RF* rheumatoid factor, *SDAI* simplified disease activity index. (DOCX 70 kb)

## Abbreviations

ACPA: Antibody against citrullinated peptides
CDAI: Clinical disease activity index
CRP: C-reactive protein
DAS28: Disease Activity Score using 28 joint counts
ESR: Erythrocyte sedimentation rate
IgG: Immunoglobulin G
IgM: Immunoglobulin M
MTX: Methotrexate
RA: Rheumatoid arthritis
RF: Rheumatoid factor
SDAI: Simplified disease activity index
TNF: Tumor necrosis factor
