# Supplementary material for: Rheumatoid factor, not antibodies against citrullinated proteins, is associated with baseline disease activity in rheumatoid arthritis clinical trials
Source: Arthritis Res Ther. 2015 Aug 26;17(1):229. doi: 10.1186/s13075-015-0736-9 (PMC4549866; doi:10.1186/s13075-015-0736-9)
Supplement: Additional file 1: Figure S1. — Disease activity at baseline in the rituximab database (IMAGE trial of early rheumatoid arthritis). Stratified analysis by the presence or absence of rheumatoid factor (RF) and of anti-citrullinated peptide antibodies (ACPA) (x-axis categories) as well as by the use, or not, of glucocorticoids. SDAI simplified disease activity index. Table S1. Disease activity indices at baseline in subgroups by RF and ACPA positivity, as well as by glucocorticoid use, or not. ACPA antibody against citrullinated peptides, CDAI clinical disease activity index, DAS Disease Activity Score, RF rheumatoid factor, SDAI simplified disease activity index. (DOCX 70 kb) [file 13075_2015_736_MOESM1_ESM.docx]

**Supplementary material to manuscript:**

*Rheumatoid factor, not antibodies against citrullinated proteins, are associated with disease activity in rheumatoid arthritis (D Aletaha, F Alasti, JS Smolen).*

**Figure S1. Disease activity at baseline in the rituximab database (IMAGE trial of early RA)** Stratified analysis by the presence and/or absence of rheumatoid factor (RF) and/or anti-citrullinated peptide antibodies (ACPA) (x-axis categories), as well as by the use, or not, of glucocorticoids.

**Table S1. Disease activity indices at baseline in subgroups by RF and ACPA positivity, as well as by glucocorticoid use, or not.**
